# An Unmanned Aerial Vehicle Indoor Low-Computation Navigation Method Based on Vision and Deep Learning

**DOI:** 10.3390/s24010190

**Published:** 2023-12-28

**Authors:** Tzu-Ling Hsieh, Zih-Syuan Jhan, Nai-Jui Yeh, Chang-Yu Chen, Cheng-Ta Chuang

**Affiliations:** Department of Intelligent Automation Engineering, National Taipei University of Technology, Taipei 10608, Taiwan

**Keywords:** indoor, unmanned aerial vehicles (UAV), obstacle avoidance, path following

## Abstract

Recently, unmanned aerial vehicles (UAVs) have found extensive indoor applications. In numerous indoor UAV scenarios, navigation paths remain consistent. While many indoor positioning methods offer excellent precision, they often demand significant costs and computational resources. Furthermore, such high functionality can be superfluous for these applications. To address this issue, we present a cost-effective, computationally efficient solution for path following and obstacle avoidance. The UAV employs a down-looking camera for path following and a front-looking camera for obstacle avoidance. This paper refines the carrot casing algorithm for line tracking and introduces our novel line-fitting path-following algorithm (LFPF). Both algorithms competently manage indoor path-following tasks within a constrained field of view. However, the LFPF is superior at adapting to light variations and maintaining a consistent flight speed, maintaining its error margin within ±40 cm in real flight scenarios. For obstacle avoidance, we utilize depth images and YOLOv4-tiny to detect obstacles, subsequently implementing suitable avoidance strategies based on the type and proximity of these obstacles. Real-world tests indicated minimal computational demands, enabling the Nvidia Jetson Nano, an entry-level computing platform, to operate at 23 FPS.

## 1. Introduction

In recent years, the development of unmanned aerial vehicles (UAVs) has gradually progressed. UAVs have applications across diverse sectors, including agriculture [1,2], architecture [3], and logistics [4]. The use of these devices within indoor settings is also gaining traction. Facilities such as factories and warehouses employ UAVs for tasks such as inventory management [5], inspection [6], surveillance [7], and intralogistics [8]. While outdoor UAVs predominantly depend on well-established and highly accurate GPSs for location detection, their functionality is compromised indoors. In such environments, infrastructure often obstructs GPS signals [9], making GPS an unreliable positioning mechanism for UAVs. Consequently, several methodologies have been introduced for indoor positioning, navigation, and obstacle detection. Historically, indoor UAVs have employed simultaneous localization and mapping (SLAM) in conjunction with lidar [10] or monocular systems [11,12]. Numerous innovative solutions have been proposed, encompassing VLC-based indoor positioning, multisensory fusion leveraging extended Kalman filters, optical flow-centric systems, and the data amalgamation of the ultrawideband (UWB) and IMU [13,14,15,16]. Notably, deep learning has recently emerged as a favored solution [17].

The main motivation for our work was to reduce both the computational demands on the embedded computer and the costs associated with device construction and upkeep. In settings such as factory inspections and intralogistics, UAVs navigate specific routes to accomplish routine tasks. While techniques such as SLAM are advanced and accurately determine position, they can be excessive in these contexts. This is due to their high computational demands and associated high equipment maintenance costs. Therefore, we introduce a streamlined approach for indoor UAV navigation that emphasizes path following and obstacle avoidance.

Our initial approach leveraged a down-looking camera with a UAV navigating using a fixed ground path and relying on real-time visual cues. This strategy, when contrasted with SLAM, is more computationally efficient. Moreover, this approach offers the advantages of lower setup and lower upkeep expenses. To enhance real-time visual path following in indoor settings, we refined the carrot-chasing algorithm and introduced the line-fitting path-following (LFPF) algorithm. For obstacle avoidance, we employed a depth camera in tandem with YOLOv4-Tiny [18]. By processing depth labels and identifying obstacle types, a UAV can effectively implement the most appropriate avoidance strategy.

By targeting indoor factories and warehouses, we developed an indoor line-tracking and obstacle-avoidance system for UAVs. The key contributions of this research are as follows:

1. We refined the carrot-chasing algorithm, enabling its indoor application with real-time vision, and maintaining an acceptable proximity to the tracking line.
2. We propose the LFPF algorithm, which not only minimizes deviations from the tracking line but also adeptly adjusts to light variations and ensures a consistent indoor flight speed.
3. We streamlined the obstacle-avoidance technique proposed by Wang [19], identifying obstacles using depth images, classifying them with YOLOv4-Tiny, and strategizing flights based on these classifications.
4. We deployed the complete system on an NVIDIA Jetson Nano entry-level embedded computer, achieving a rate of 23 FPS.

## 2. Related Work

### 2.1. Path-Following Algorithm

Path planning is crucial for UAVs. Given that the forces exerted on a UAV during flight differ significantly from those on ground vehicles, numerous UAV-specific path-following algorithms have been proposed. Brandao [20] presented a vision-based line-following strategy designed for autonomous UAVs in agriculture. The nonlinear path following the controller design ensures system stability. Tkachev [21] addresses the challenge of UAV path tracking at a specified altitude, representing the target path as a planar curve. Silva [22] introduced an innovative UAS approach for precise navigation over complex oil and gas pipelines using image processing and a convolutional neural network (CNN). Sujit [23] analyzed five readily applicable path-following algorithms for UAVs. The study revealed that each algorithm offers a distinct balance between control effort and path accuracy, contingent on the intended use. Notably, the carrot-chasing algorithm, pure pursuit, line-of-sight-based path following (PLOS), and linear quadratic regulator-based path following (LQR) require less control effort than the vector field path-following algorithm; however, these algorithms surpass the nonlinear guidance law in terms of accuracy.

Given its efficiency in controlling effort and its high accuracy in path tracking, the carrot-chasing algorithm was selected as our path-following method. This algorithm establishes a virtual target point (VTP) along the path, guiding the UAV to pursue it.

As shown in Algorithm 1, $W_{i}$ and $W_{i+1}$ are the two weight points on the path. The midpoint of the two weight points was set as the VTP, and point p was the position of the UAV. This algorithm outputs u, which is used to control the yaw rate.

Originally designed for outdoor use, this algorithm enables a UAV to execute turns with a broader radius. Furthermore, the UAV prefers the entire path in this algorithm, with GPS signals facilitating course corrections if significant deviations occur. However, indoor spaces present constraints. The use of a down-looking camera to capture the path also restricts the UAV’s maximum deviation. As such, we refined the carrot-chasing algorithm to better fit this context. Moreover, this enhanced version serves as the benchmark for tracking displacement, against which we assessed our proposed tracking algorithm, LFPF.

**Algorithm 1** Carrot-chasing algorithm
Input: $W_{i}=\left(x_{i},\;y_{i}\right),\;{\;W}_{i+1}=\left(x_{i+1},y_{i+1}\right),\;\;p=\left(x,\;y\right),\;\psi$ Output: u
1

while true do

2

$\;\;{\;\;W}_{i}=\left(x_{i},\;y_{i}\right)$

3

${\;\;\;\;W}_{i+1}=\left(x_{i+1},y_{i+1}\right)$

4

$\;\;\;\;p=\left(x,\;y\right)$

5

    ψ=current angle

6

${\;\;\;\;R}_{u}=\left\|W_{i}-p\right\|,\;\theta =atan2(y_{i+1}-y_{i},\;x_{i+1}-x_{i})$

7

${\;\;\;\;\theta }_{u}=atan2\left(y-y_{i},\;x-x_{i}\right)$


$,\;\beta =\theta -\theta_{u}$

8

$\;\;\;\;R=\sqrt{R_{u}^{2}-{\left(R_{u}\sin\left(\beta \right)\right)}^{2}}$

9

$\;\;\;\;s=\left(x_{t}^{\prime },\;y_{t}^{\prime }\right)=(\left(R+\delta \right)cos\theta +x_{i},\;\left(R+\delta \right)sin\theta +y_{i})$

10

${\;\;\;\;\psi }_{d}=atan2\left(y_{t}^{\prime }-y,\;x_{t}^{\prime }-x\right)$

11$\;\;\;\;u=\kappa (\psi_{d}-\psi )$, with κ > 012

End



### 2.2. Obstacle Detection Method Based on Vision

Object detection and avoidance empower UAVs to swiftly circumvent unexpected obstacles, enhancing navigational safety. A cost-effective obstacle detection method [24] uses DroNet [25] to assess crash probability, halting the UAV preemptively. While economically efficient in terms of its computation and hardware, DroNet is subject to specific environmental conditions. For accurate operation, the UAV must follow a strict trajectory or the precision of DroNet must be diminished.

Wang [19] presented a solution amalgamating deep learning with a depth camera capable of discerning not only the proximity of obstacles but also their characteristics. Thus, a UAV can craft an avoidance strategy tailored to an obstacle’s attributes. While Wang [19] used YOLOv3 [26] for object detection, this approach was resource intensive. To address the issue of fast-moving drones, Liu [27] pruned the YOLOv4 model to increase the processing speed. Simultaneously, a special augmentation technique was implemented to improve the detection accuracy of small drones.

The YOLOv4-Tiny [28] method, an offshoot of YOLOv4, combined with NVIDIA TensorRT [29], a C++ library optimized for NVIDIA GPUs, boosts object detection performance [18]. Converting a deep learning model into the TensorRT format can expedite its inference on NVIDIA GPUs. Jkjung-Avt [30] proposed a method for transitioning from the Darknet framework to the TensorRT format. In our approach to obstacle detection, we integrated YOLOv4-Tiny in TensorRT format with a depth camera.

## 3. Method

The UAV is outfitted with a downward-facing camera and a forward-facing depth camera. For path tracking, the device gleans displacement data from the downward-facing camera and harnesses either the enhanced carrot-chasing algorithm or the LFPF algorithm to adjust its orientation. In tackling obstacle avoidance, the depth of an impediment can be ascertained by applying thresholds to the depth image. Concurrently, the nature of the obstacle is derived from the RGB image using YOLOv4-Tiny. Ultimately, the strategy for avoidance is chosen by considering both the depth and type classifications of the obstacles.

Our proposed method can be separated into two parts: tracking and obstacle avoidance. The first and second parts of this section introduce the hardware and the overall workflow of our method. The tracking and obstacle avoidance methodologies are subsequently explained individually.

### 3.1. Hardware

**Figure 1:**
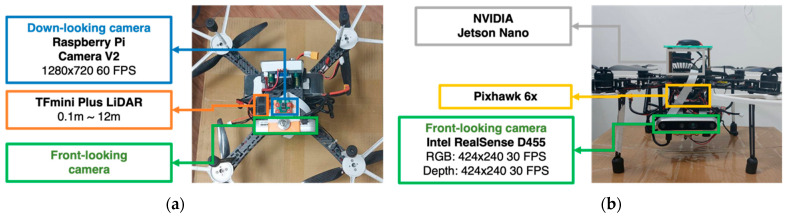
Installation of the hardware in (**a**) the bottom view of the UAV and (**b**) the front view of the UAV.

**Figure 2:**
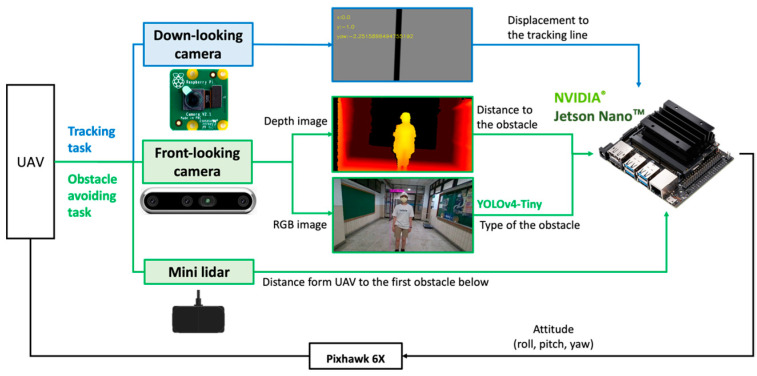
Control relationship between hardware.

The hardware and its relationships are shown in Figure 1. Table 1 lists the specifications of the drone hardware components. Figure 2 shows the installation of the hardware. This study utilized Pixhawk 6X as the flight controller and an Nvidia Jetson Nano B01 as the embedded computer for the UAV.

To facilitate tracking and obstacle avoidance, three supplementary sensors were incorporated into the UAV. First, the Raspberry PiCamera V2 served as the downward-facing camera, enabling the UAV to discern its displacement from the tracking line. Second, an Intel Realsense D455 was utilized as the forward-facing camera to capture both the depth and RGB images of obstructions. Finally, the TFmini Plus LiDAR was integrated to measure the vertical distance between the UAV and either the floor or any prominent obstacle beneath.

### 3.2. Implementing Workflow

**Figure 3:**
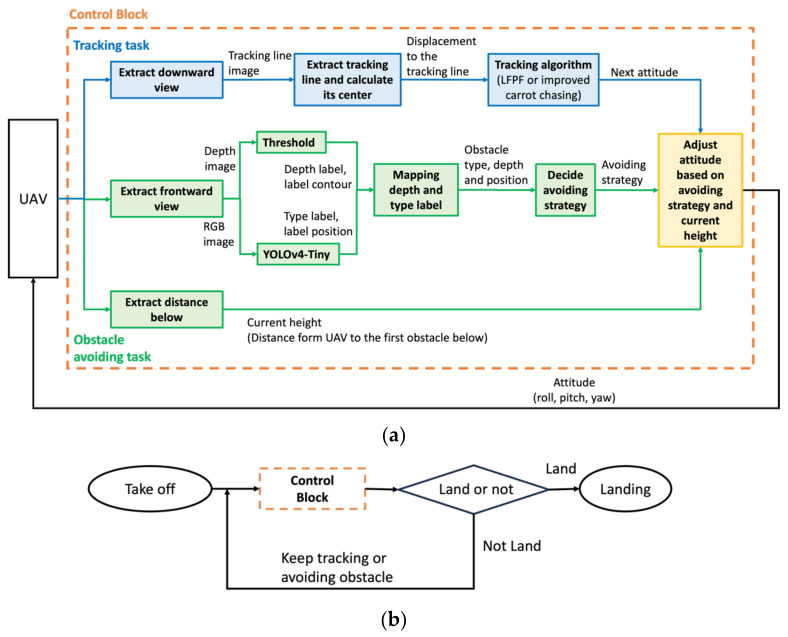
(**a**) Implementation architecture; (**b**) Implementation flow chart

The implementation architecture is shown in Figure 3a. For path tracking, UAV ground displacement data are obtained from a downward-facing camera, and either the enhanced carrot-chasing algorithm or the LFPF algorithm is used to adjust the orientation of the UAV. In tackling obstacle avoidance, the depth of an impediment can be ascertained by applying thresholds to the depth image. Concurrently, the nature of the obstacle is derived from the RGB image using YOLOv4-Tiny. After mapping the depth and type classifications of the obstacles, the strategy for avoidance is chosen. The rangefinder provides the distance between the UAV and the ground or the obstacle below it. Ultimately, a new attitude is generated by considering the tracking, obstacle avoidance, and current height results. Figure 3b uses the control block in Figure 3a to demonstrate the implementation flow chart.

### 3.3. Path Following

We refined the carrot-chasing algorithm and introduced the LFPF algorithm. For efficiency validation, we initially tested both algorithms in a simulated environment, followed by real-flight verification.

#### 3.3.1. Simulation Environment

**Figure 4:**
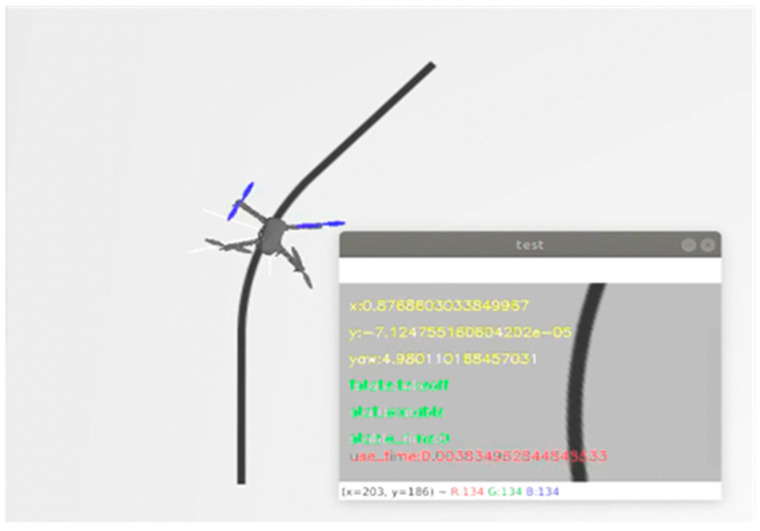
UAV in the simulation environment with the view from the ROSCAM at the lower left corner of the figure.

We constructed our simulation environment on Ubuntu 18.04 LTS using the VMware Workstation 16 Player. This environment included ROS Kinetic, Gazebo, and Ardupilot SITL. We created the tracking line within the simulation using Blender 4.0.2 software. A ROS camera was positioned beneath the virtual UAV to simulate a real-world down-looking camera. Figure 4 shows a UAV flying in the simulation environment.

#### 3.3.2. Improved Carrot-Chasing Algorithm

In Algorithm 2, the improved carrot-chasing algorithm, roll angle, and pitch angle are generated according to the constants *c*_1_ and *c*_2_, pitch, and output of the carrot-chasing algorithm, u. Physically, pitch refers to the velocity of the UAV, and *u* refers to the yaw angle at which the UAV should turn to reach the VTP. In addition to *u*, the improved carrot-chasing algorithm uses roll and pitch angles to control the UAV. The larger the yaw angle is, the larger the roll angle needed to provide centripetal force, and the smaller the pitch angle needed to reduce the speed of the turn. Owing to the centripetal force, the UAV did not drift away from the tracking line.

The $W_{i}$, $W_{i+1}$, two way-points, and *p,* the position of the UAV, must be defined to apply this algorithm. To obtain the $W_{i}$ and $W_{i+1},$ the following processes must be performed for each frame: after binarizing the image, 20 pixels were retained near the boundary. The centers of the remaining two parts were labeled. The upper panel represents $W_{i+1}$ and the lower panel represents $W_{i}$. The center of the image obtained by the down-looking camera is represented by *p*.

**Algorithm 2** Improved Carrot-Chasing Algorithm
Input: $W_{i}=\left(x_{i},\;y_{i}\right),W_{i+1}=\left(x_{i+1},\;y_{i+1}\right),\;p=\left(x,y\right),\;\psi$ Output: u, roll, pitch
1

while true do

2

$\;\;{\;\;W}_{i}=\left(x_{i},\;y_{i}\right)$

3

${\;\;\;\;W}_{i+1}=\left(x_{i+1},y_{i+1}\right)$

4

$\;\;\;\;p=\left(x,\;y\right)$

5

    ψ=current angle

6

$\;\;\;\;u=Carrot\;Chasing\;algorithm(W_{i},W_{i+1},p)$

7

$\;\;\;\;roll=c_{1}\times pitch\times {(e}^{sin\left(u\right)}-1)$

8

$\;\;\;\;pitch=c_{2}\times pitch\times (1-\cos\left(u\right))$

9

End



#### 3.3.3. LFPF Algorithm

The enhanced carrot-chasing algorithm exhibited strong performance within the simulation environment. Nonetheless, during the actual flight, tracing lines near the image boundary were not discerned due to fluctuations in lighting. Additionally, the UAV had to sustain a high speed to correct any misalignment, posing a safety concern indoors. As a solution, we introduce the line-fitting path-following (LFPF) algorithm.

**Figure 5:**
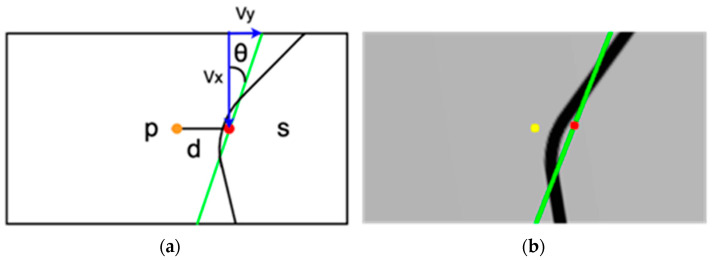
(**a**) The concept of the LFPF algorithm; (**b**) the curve captured by the ROSCAM with a fitted line, point s (red dot), and p (yellow dot).

This approach involves line-fitting on the binarized tracking line. Subsequently, the M-estimator’s least square distance was used to gauge the slope and intercept of the drawn line. As shown in Figure 5, the green line represents the fitted line, *s* is the midpoint of the fitted line, and *p* is the center of the image, which refers to the position of the UAV.

As shown in Algorithm 3, the slope and displacement from p to s generate the yaw rate, roll angle, and pitch angle. These are subsequently used to control the UAV. The fourth line in Algorithm 3 gives the relationship between the x_i_ and the roll. When the error was close to zero, the roll changed significantly. As the error increases, the roll changes slightly. We observed a time lag between the control command of the UAV to change its flight direction and the actual flight direction. The fourth line allows the precise adjustment of the *x*-axis displacement and prevents the UAV from surging in the other direction.

**Algorithm 3** Line-Fitting Path-Following Algorithm.
Input: $p=\left(x,y\right),\;s=\left(x_{i},y_{i}\right)$

Output: roll, pitch
1

while true do

2

${\;\;\;\;v}_{x},v_{y},x_{i},y_{i}=fitLine(all\;points\;of\;line)$

3

$\;\;\;\;\theta =yaw=\frac{180}{\pi }\times {tan}^{-1}\left(\frac{v_{y}}{v_{x}}\right)$

4

$\;\;\;\;roll=c_{1}\times \left(\frac{1}{1+e^{\frac{\left(x_{i}-x\right)}{80}}}-0.5\right)$

5

$\;\;\;\;pitch=c_{2}\times \left|sin\theta \right|+b$

6

End



$v_{x},v_{y},x_{i},y_{i}$ is the result of the line-fitting algorithm using all points of the line. $\frac{v_{y}}{v_{x}\;}$ is the slope of the fitted line, and $\left(x_{i},\;y_{i}\right)$ is the midpoint of the line.

#### 3.3.4. Evaluation

To evaluate the algorithms, we designed two line patterns. The first entails a 10 m straight tracking line, while the second features a bend with 1 m straight lines preceding and following it. This bend has a curve radius of 1.5 m and a rotation angle of 45°.

The evaluation of the algorithms hinged on two primary factors: speed and accuracy. We monitored the pitch because it directly affects the UAV’s speed. For gauging accuracy, we measured the UAV’s displacement from the tracking line along the *x*-axis, noting this as an error. Throughout the flight, we charted the variations in these two parameters. For the *x*-axis error, both the range and mean values were documented. The mean value computation was grounded on Equation (1), where *x* denotes the error at time *t*, and *n* signifies the cumulative count of *x*.

(1) $$mean=\frac{\sum_{t=0}^{n}x}{n}$$

### 3.4. Obstacle Avoidance

In this study, we utilized an Intel Realsense D455 as the forward-facing camera to capture a real-time image stream in front of the UAV, comprising both RGB and depth images. Based on these images, the UAV can determine an appropriate obstacle avoidance strategy.

#### 3.4.1. Depth Detection

**Figure 6:**
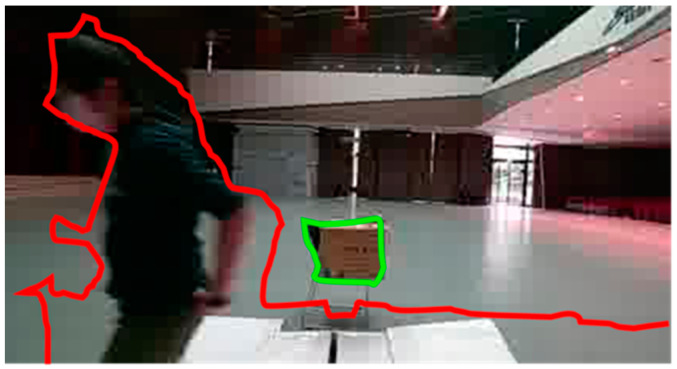
“Near” (red) and “far” (green) labels for the image.

Obstacles are categorized as “near” or “far” based on their distance from the UAV. Images were binarized using threshold values of 1.2 m and 2 m to classify these obstacles. Notably, if the 2 m threshold contour encompasses the 1.2 m threshold contour, it is labeled “near”. The contours, along with their distance labels, are processed as depicted in Figure 6.

#### 3.4.2. Obstacle Detection

We employed YOLOv4-Tiny to identify obstacle types. Our classification includes “people”, “boxes”, “carts”, and “stackers”, which are common entities in indoor factory settings. To efficiently run the model on an NVIDIA Jetson Nano with a satisfactory FPS, we converted the model from darknet to a TensorRT format. This transformation reduced the number of layers from 77 to 50 due to TensorRT’s ability to merge the convolution layer with the batch normalization layer and omit some route layers. The resulting model provides both the type label and the center position of the detected obstacles.

#### 3.4.3. Mapping Obstacle Type to Depth

To correlate the type and depth labels, each depth contour is examined to determine whether it contains the center of an obstacle. If a contour encloses the center of a recognized type, it adopts that type label. Conversely, if a depth contour does not correspond to any obstacle center detected by YOLOv4-Tiny, it is labeled “Unidentified”. In cases where multiple centers are contained within a single contour, the obstacle type is determined in the sequence of “people”, “boxes”, “carts”, and “stackers”.

#### 3.4.4. Avoiding Strategy

**Figure 7:**
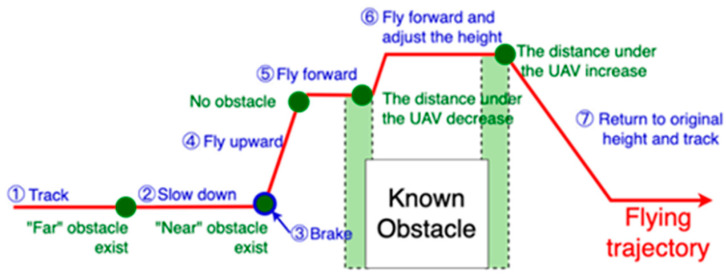
Explanation of steps for a UAV to avoid known obstacles.

As shown in Table 2, by knowing the obstacle type and distance from the UAV, the corresponding obstacle avoidance strategy can be chosen. Priorities I to IV are simultaneously set for several contours. A smaller value indicates a higher priority. Figure 7 shows how the UAV flies upward to avoid known obstacles in priority III.

## 4. Results

The experiment consisted of two sections. First, we conducted an experiment to evaluate the speed and accuracy of the improved carrot-chasing and LFPF algorithms in a simulation environment. Second, to verify our obstacle-avoiding strategy, actual flights with tracking and obstacle-avoiding tasks were performed.

### 4.1. Tracking Task in the Simulation Environment

To evaluate the speed and accuracy of the algorithm, the pitch angle and *x*-axis error of the UAV were recorded. The parameters used in this experiment are listed in Table 3.

#### 4.1.1. Straight Line

**Figure 8:**
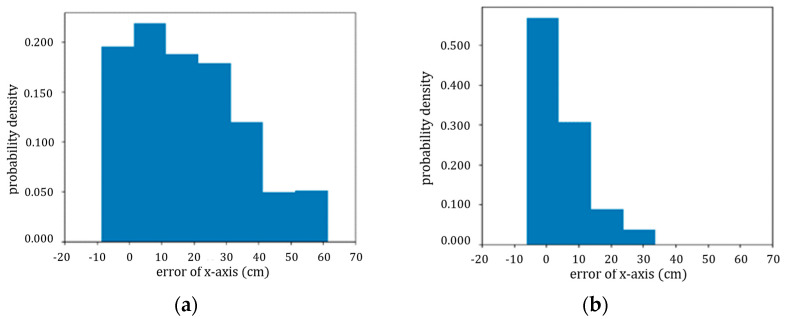
(**a**) PDF of the *x*-axis error in the improved carrot-chasing algorithm; (**b**) PDF of the *x*-axis error in the LFPF.

These algorithms have distinct initial pitch angles because the concept of each algorithm varies. The initial pitch angle of the improved carrot-chasing algorithm is −1°, which is large enough for the UAV to chase the target point, whereas the initial pitch angle of the LFPF algorithm is only −0.19°. Figure 8 shows the probability density function (PDF) of the *x*-axis error of the UAV during a straight-line flight. For the improved carrot-chasing algorithm, the maximum *x*-axis error was 60 cm, and the mean *x*-axis error was 18.59 cm. In contrast, for the LFPF algorithm, the maximum *x*-axis error was 30 cm, and the mean *x*-axis error was 6.17 cm. The values of the LFPF algorithm are more concentrated than those of the improved carrot-chasing algorithm. In the straight-line test, the LFPF algorithm exhibited better accuracy.

#### 4.1.2. Bend

**Figure 9:**
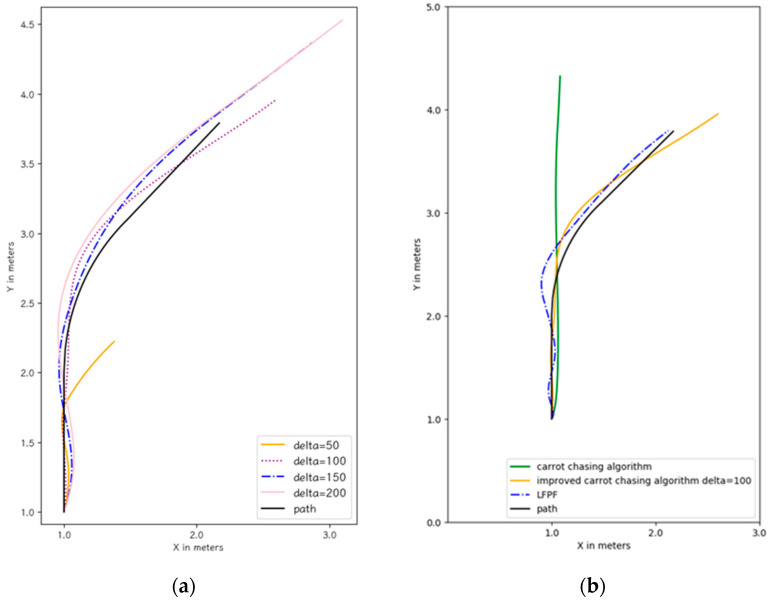
(**a**) Flight trajectories of carrot-chasing using different deltas; (**b**) Flight trajectories of different algorithms.

Since the flight trajectories of the improved carrot-chasing algorithm vary with δ in the bend, we optimized the value of δ with the best sensitivity to the displacement at δ = 100 (Figure 9a). Thus, the improved carrot-chasing algorithm was compared using δ = 100. We also tried to optimize the carrot-chasing algorithm by adjusting δ. However, the carrot-chasing algorithm did not perform well irrespective of the value of δ.

A comparison of the bend-tracking task results of the carrot-chasing algorithm, improved carrot-chasing algorithm, and LFPF algorithm are shown in Figure 9b. Both the improved carrot-chasing algorithm and the LFPF algorithm smoothly performed the turn.

### 4.2. Actual Flight

In the actual flight experiment, we verified the tracking task with the LFPF algorithm as well as the obstacle-avoiding task with various obstacles.

#### 4.2.1. Tracking Task

**Figure 10:**
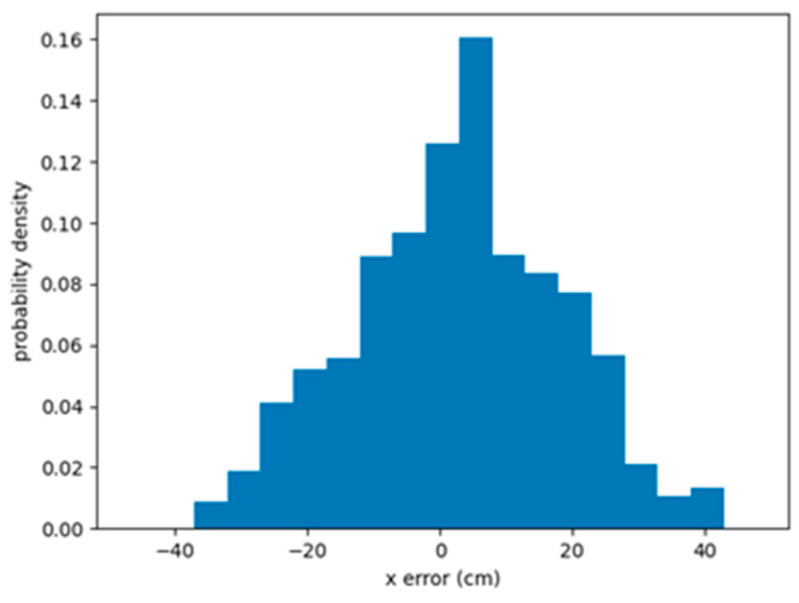
PDF of the *x*-axis error of the actual flight.

With the parameter list shown in Table 2, the UAV can perform the tracking task using the LFPF algorithm during an actual flight. The PDF of the *x*-axis error over 10 flights of the 10 m straight line is shown in Figure 10. The UAV could maintain its displacement within ±40 cm, with a mean displacement of 13.30 cm.

#### 4.2.2. Obstacle Avoidance Task

**Figure 11:**
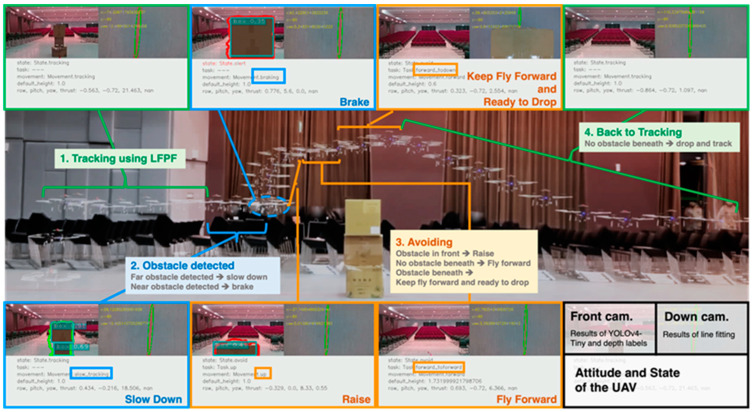
Flying state of the UAV while avoiding “boxes”. Front-looking and down-looking scenes are also shown for each state.

Three obstacle types—”boxes”, “chairs”, and “people”—were evaluated in the obstacle avoidance task, with “chairs” symbolizing the unidentified obstacle. To confirm the UAV’s predicted response, we documented both its frontward and downward views, as well as its evasion status. Figure 11 shows both the flight trajectory of the UAV while it was avoiding the “boxes” and our video record. The YouTube video link to the results of this experiment is https://youtu.be/Efy-fQ2ab28 (accessed on 14 December 2023).

**Figure 12:**
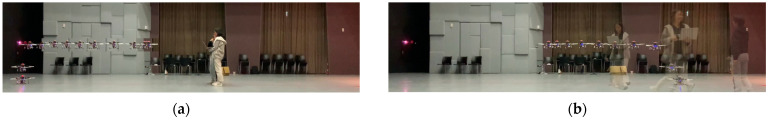
(**a**) The UAV detected people and hovered; (**b**) The UAV detected people who left and kept flying.

**Figure 13:**
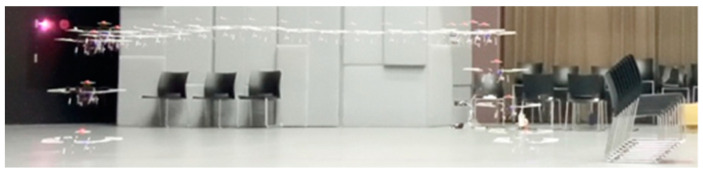
The UAV detected the unidentified obstacle and landed.

The trajectory is the same as that shown in Figure 8. Figure 12 shows the UAV flight trajectory while avoiding people. The UAV hovered until it left the tracking line. As shown in Figure 13, the UAV detects an unidentified obstacle and lands.

#### 4.2.3. Computation

The system was implemented on an NVIDIA Jetson Nano, which was the primary embedded computer.

With the YOLOv4-Tiny compressed, the computational speed of the entire system could reach 23 FPS. Table 4 compares the FPS, GPU utility, CPU utility, and RAM usage during execution before and after the YOLOv4-tiny is compressed.

### 4.3. Comparison of Our Paper with Existing Solutions

The existing papers on obstacle avoidance strategies are not the same; we select important items for comparison. Table 5 shows the comparison between our proposed method and other solutions.

## 5. Discussion and Future Work

This study proposes a UAV tracking and obstacle avoidance system designed specifically for indoor flights. Bypassing the need for optical flow or beacons, this system enables efficient indoor UAV navigation with a reduced computational load.

For tracking, we evaluated both the improved carrot-chasing and LFPF algorithms on straight and curved paths. The test results yielded accurate and speedy data. Based on the pitch angle, the UAV’s speed when using the LFPF is approximately 20% of that when using the improved carrot-chasing algorithm. Consequently, the LFPF algorithm results in a slower, more stable speed, enhancing the safety of indoor flights.

Experiments in both simulated and real-world settings revealed that transitioning the tracking task using the LFPF algorithm from a simulation to an actual environment necessitates only adjustments to coefficients *c*1 and *b*: *c*1 shifted from 1.2 to 3.2, and *b* from 0.14 to 0.72.

For obstacle avoidance, we employed a depth camera to capture depth and RGB images, facilitating the identification of obstacle depths and types. The obstacle recognition process in this paper employs YOLOv4-Tiny, a network known for its advantages of being lightweight and executing quickly. After undergoing TensorRT conversion, the execution speed of the Jetson Nano platform improved. Additionally, this paper is designed for applications in a factory setting where the types of objects to be recognized are relatively fixed. The ability to rapidly detect the presence of obstacles is a crucial consideration in this context, as YOLOv4-Tiny is a suitable choice for this paper’s application. Although YOLOv4-Tiny sometimes detects obstacles where none exist, this system mitigates such errors by assessing the obstacle’s depth and utilizing type information to refine the chosen avoidance strategy. The reliability of YOLOv4-Tiny increases when the UAV is near an obstacle.

The trajectory lines assessed in this study represent fundamental patterns, gauging the algorithms’ adaptability to straight and curved paths. However, to ensure the practical utility of UAVs in indoor settings, in the future, the processing of downward view images and tracking algorithms should be improved by considering more complicated tracking line patterns and tracking line intersections.

Additionally, the current avoiding method, a default upward motion, requires the application scenario to either have a high ceiling or not heap the obstacles high. Therefore, refining the avoidance strategy by accounting for the prevailing safety space that could guide the UAV toward a more discerning avoidance strategy is another issue for future work.

## 6. Conclusions

This study focused on tracking and obstacle avoidance tasks for indoor UAVs. The proposed LFPF indoor line-tracking algorithm was juxtaposed with an enhanced carrot-chasing algorithm in a simulated environment. The displacement of the LFPF on the *x*-axis was consistently closer to zero than that on its counterpart. The LFPF algorithm had an average x-error of 6.17, which is 12.42 cm less than that of the improved carrot-chasing algorithm. In curved scenarios, both algorithms execute smooth turns, with the LFPF displaying minimal oscillation post-turn.

During obstacle avoidance trials, the UAV maintained an *x*-axis error within ±40 cm, adapting its avoidance strategy based on the detected obstacle type and distance. The system was processed at 23 FPS on an Nvidia Jetson Nano. Nevertheless, real-world applications might present more intricate tracking line configurations, and space constraints can differ. A single avoidance strategy might not suffice universally, highlighting areas for future research focus.

## Figures and Tables

**Table 1:** List of hardware components.

Hardware	Specification
Pixhawk 6X	FMU Processor: STM32H753 (32 Bit Arm^®^ Cortex^®^-M7, 480 MHz, 2 MB flash memory and 1MB RAM).
Jetson Nano	128-core NVIDIA Maxwell™ GPU Quad-core ARM A57 @ 1.43 GHz 4 GB 64-bit LPDDR4|25.6 GB/s
Raspberry Pi Camera V2	1280 × 720 60 FPS
Intel RealSense D455	RGB: 424 × 240 30 FPS Depth: 424 × 240 30 FPS
TFmin Plus LIDAR	0.1 m–12 m

**Table 2:** Depth and type of obstacle and the corresponding avoidance strategy.

Obstacle Contour		Obstacle Type		
		Unidentified	People	Known Obstacle (Boxes, Carts, or Stackers)
Depth	Near	I. Hover and notify the ground station.	II. Hover and alarm the people until they leave.	III. Fly upward to avoid the obstacle.
	Far	IV. Slow down.		

**Table 3:** Parameter List for Different Tracking Algorithms.

Simulation Environment		Actual Flight
Improved CC	LFPF SIM	LFPF
$c_{1}$ = 0.0013	$c_{1}$ = 3.2	$c_{1}$ = 1.2
$c_{2}$ = 0.016	$c_{2}$ = 0.01	$c_{2}$ = 0.01
*δ* = 100	*b* = 0.14	*b* = 0.72

**Table 4:** Comparison of the Darknet and TensorRT formats.

Format	FPS	GPU Utility (%)	CPU Utility (%)	RAM Usage
Darknet	16–17	77–99	58–100	2.7 GB
TensorRT	24–25	75–99	30–70	3.3 GB

**Table 5:** Comparison with existing solutions.

Items	Our Study	[31]	[32]
Real-world Experiment	Yes	Yes	No, combining the simulation environment with the real-world
Method	depth threshold + YOLOv4	LiDAR + SLAM	depth threshold + YOLOv3
Dynamic obstacle?	Yes	No	Yes
Computational Platform	Jetson Nano (Cheaper)	Jetson TX2	GPU:NVIDIA GTX1080;
Indoor/Outdoor	Specific for Indoor Factory	Indoor + unknown environment	Outdoor Specific for farm
Advantage	Human	unknown environment, building map, LTE Communication	Better navigating speed:5 m/s

## Data Availability

Data available on request from the authors.
